# Perils and Pitfalls Regarding Differential Diagnosis and Treatment of Primary Cutaneous Anaplastic Large-Cell Lymphoma

**DOI:** 10.1100/tsw.2011.80

**Published:** 2011-05-05

**Authors:** Michael D. Diamantidis, Athena D. Myrou

**Affiliations:** Department of Haematology, First Propedeutic Department of Internal Medicine, Aristotle University of Thessaloniki, AHEPA Hospital, Thessaloniki, Greece

**Keywords:** primary cutaneous anaplastic large-cell lymphoma (PC-ALCL), T–non-Hodgkin lymphoma (T-NHL), CD30^+^ T-cell lymphoproliferative disorder (PCLPD), immunohistochemistry, systemic anaplastic large-cell lymphoma (ALCL), anaplastic lymphoma kinase (ALK), cutaneous lymphocyte antigen (CLA), epithelial membrane antigen (EMA), multifocal lesions, disseminated disease, differential diagnosis, treatment

## Abstract

Primary cutaneous anaplastic large-cell lymphoma (PC-ALCL), belonging to the CD30+ T-cell lymphoproliferative disorders (PCLPDs), is a rare T-cell lymphoma, presenting on the skin and characterized by very good prognosis and response to treatment in the majority of cases. Nevertheless, PC-ALCL must be distinguished from secondary skin lesions in systemic ALCL, which confer a poor prognosis, and other CD30+ PCLPDs, reactive conditions, or borderline cases. Given their rarity and heterogeneity, these entities represent diagnostic and therapeutic challenges, thus requiring a multidisciplinary approach and expertise to ensure appropriate diagnosis and management. There are several perils and pitfalls that exist regarding the differential diagnosis, the possible progression, and the treatment of PC-ALCL. Careful staging, correlation of clinical findings with histopathology and immunopathology, and thorough follow-up are essential in order to achieve a correct diagnosis and proper treatment of the disease.

## INTRODUCTION

Primary cutaneous CD30+ T-cell lymphoproliferative disorders (PCLPDs) are the second most common type of cutaneous T-cell lymphomas, accounting for approximately 30% of all cases[1]. These disorders include the clinically benign conditions lymphomatoid papulosis (LyP), primary cutaneous anaplastic large-cell lymphoma (PC-ALCL), and borderline cases. The incidence of PC-ALCL among other types of peripheral T-cell non-Hodgkin lymphomas is 1.7% and peaks in the sixth decade of life, with a median age of 61 years[2,3]. Hence, PC-ALCLs are uncommon. Interestingly, PC-ALCL accounts for 9% of all cutaneous T-cell lymphomas. Using conservative measures, 5-year disease-free survival rates are >90%.

However, a subset of PC-ALCL patients with worse treatment and survival outcome having extensive limb disease has been reported. Moreover, PC-ALCL has rarely been described in the pediatric population. It can also affect young adults[4].

PC-ALCL is a lymphoma composed of large cells with an anaplastic, pleomorphic, or immunoblastic cytomorphology and expression of CD30 antigen by the majority of the tumor cells. PC-ALCL has a wide clinical range, from small self-regressing papules in the majority of the cases, to widespread disseminated tumors progressing to nodal participation and systemic involvement. Thus, correct classification of CD30+ lymphomas is a confusing process, depending heavily on clinical features[5]. Staging and observation are indicated for PC-ALCL, along with a period of watchful waiting to allow for self-regression. Nevertheless, at presentation, the natural history of the disease may not be obvious and may be altered by molecular and genetic modifications in the cellular components and their interactions with the host and the microenvironment.

The clinical manifestations, morphology, immunophenotype, molecular characteristics, differential diagnosis, and treatment of PC-ALCL are highlighted in this review. Special focus is given to the perils and pitfalls that exist concerning the treatment and the differential diagnosis of PC-ALCL.

## CLINICAL MANIFESTATIONS

Although the head and extremities have been reported to be sites with predilection to PC-ALCL[6], there is a lack of information in the literature concerning the distribution of these rare neoplasms in particular anatomical sites of the upper or lower limbs. Careful staging, immunohistochemistry, and thorough clinical follow-up remain the most reliable methods to confirm a PC-ALCL diagnosis. Most patients with PC-ALCL present with solitary, firm, often ulcerated or localized skin tumors[6]. The lesions may be single and large, or several and fused, or clustered. Less often, PC-ALCL might present as multicentric tumors or nodules, which can coexist with papules of LyP. Furthermore, localized involvement in one anatomic region with multiple lesions occurs less frequently in comparison to localized involvement with one lesion[5]. Multifocal cutaneous disease is defined as the involvement of two or more anatomic areas or an isolated lesional area exceeding 15 × 15 cm. Generalized or multifocal lesions exist in about 20% of the patients[4]. The tumors occur on normal skin without previous patches or plaques, and are usually asymptomatic.

Patients with disseminated skin disease are prone to develop extracutaneous involvement and might benefit from systemic chemotherapy. Extracutaneous involvement is traced in 5–10% of the patients at presentation and commonly occurs with distant nodal involvement[3]. PC-ALCL lesions are known to regress spontaneously in some cases.

## MORPHOLOGY, IMMUNOPHENOTYPE, AND MOLECULAR CHARACTERISTICS

PC-ALCL is characterized by cohesive sheets of large cells with an anaplastic, pleomorphic, or immunoblastic morphology. The tumor cells have irregular nuclei, invade the skin appendages, and extend into the subcutaneous fatty tissue, infiltrating the dermis and subcutis. Mitoses, apoptotic bodies, vascular invasion, and variable inflammatory infiltrates are also noted. It is defined by CD30 expression of at least 75% of the large lymphoid cells. Multinucleated Reed-Sternberg–like forms are seen, and cells with horseshoe or wreath-like configurations are also identified. The atypical cells show an activated CD4+ T-helper cell phenotype with variable loss of T-cell markers and expression of cytotoxic proteins, such as granzyme B, TIA-1, and perforin, in about half of the cases. CD25 and HLA-DR can also be found. CD8+ T-cell phenotype, null CD4-CD8- phenotype, and co-expression of CD56 and CD30 are extremely rare in PC-ALCL[4].

PC-ALCLs more frequently express the cutaneous lymphocyte antigen (CLA), but lack the epithelial membrane antigen (EMA). Anaplastic lymphoma kinase (ALK) expression is also negative in most cases of PC-ALCL. Molecular studies have shown that nearly all cases of PC-ALCL are of clonal origin. Research using array-based comparative genomic hybridization has revealed chromosomal imbalances in 40% of all PC-ALCL cases[5].

## DIFFERENTIAL DIAGNOSIS – PERILS AND PITFALLS

The clinician must be aware of several perils and pitfalls that exist regarding the differential diagnosis and the possible progression of PC-ALCL. PC-ALCL may present as localized or multifocal cutaneous disease, or may progress to widespread systemic disease. The term ALCL is used to describe the systemic form of PC-ALCL, although it is more uncommon than localized PC-ALCL. Systemic ALCL is an aggressive lymphoma that may secondarily involve the skin, in addition to other extranodal sites. These two forms of ALCL are distinct entities with different clinical and biological features. Nevertheless, both types share the similar histology with cohesive sheets of large lymphoid cells expressing the Ki-1 (CD30) molecule[4]. Interestingly, the distinction between the two forms of ALCL might be an oversimplification[7]. It is not yet well defined which cases of PC-ALCL will disseminate to lymph nodes or progress to a systemic lymphoma and why. Moreover, the molecular events during this transformation have not been fully elucidated. Systemic ALCL primarily involves lymph nodes, even though extranodal sites might be implicated.

Systemic ALK+ ALCLs have improved prognosis in comparison to ALK- ALCLs, even though chemotherapy is strongly suggested to patients with both subtypes[2]. Approximately 20% of patients with systemic ALCL have skin involvement[8]. Systemic ALK+ ALCL is more frequent in men younger than age 35, whereas systemic ALK- ALCL is more common in older patients, with a median age of 61 years. Furthermore, the extranodal sites in ALK- ALCL include bone marrow, bone, subcutaneous tissue, and the spleen, while in ALK+ ALCL, the most frequent sites are skin, bone, bone marrow, liver, and lung[9]. However, in the majority of patients, systemic ALCL at presentation is advanced stage III or IV disease with lymphadenopathy, B symptoms, and a rapidly progressive clinical course[5].

Nuclear matrix protein (NMP)-ALK is a nuclear fusion protein that is not expressed in normal lymphocytes and, when present, is transforming. The ALK protein is activated most frequently through the nonrandom t(2;5) chromosome translocation, resulting in fusion of the nucleophosmin (NPM) gene located at 5q35 to 2p23 and encoding the receptor tyrosine kinase ALK[10]. In contrast to systemic ALCL, PC-ALCLs seldom carry the t(2;5) translocation. ALK positivity in ALCL indicates abnormal expression of the ALK protein and, therefore, rearrangement of the ALK gene. The ALK gene is not rearranged in ALK negative cases[10].

ALK expression in the skin in the majority of cases indicates systemic disease and is a warning to look for systemic involvement because it is infrequent in PC-ALCL[8]. However, rare cases of ALK+ PC-ALCLs have been described. Conversely, ALK negativity in the skin does not exclude systemic disease. Reliable criteria are not currently available to distinguish PC-ALCL from systemic ALCL (ALK-) involving the skin. In addition, like ALK, EMA expression is usually present in systemic ALCL with skin involvement, rather than in PC-ALCL[3,8,11].

Correlation of clinical findings with histopathology and immunopathology (stains for ALK protein, EMA, and CLA) are essential to achieve a correct diagnosis. It has not been elucidated why ALK+ expression is a favorable tumor marker in systemic ALCL, but is not present in PC-ALCL, which has an excellent 5-year overall survival (OS) rate[7]. In addition, both PC-ALCL and systemic ALCL share large lymphoid neoplastic cells expressing CD30, while the ALK- PC-ALCL has a favorable prognosis in contradiction to the poor outcome of ALK- systemic ALCL. To explain the above inconsistency, it has been proposed that expression of the apoptotic protein Bax and CD30-ligand–mediated cytotoxicity may be associated with spontaneous regression in PC-ALCL[12]. Nevertheless, extensive research is needed to clarify the above-mentioned discrepancy.

The diagnosis of PC-ALCL requires the exclusion of other cutaneous infiltrates characterized by CD30 expression[13]. These conditions include reactive and neoplastic diseases, such as arthropod bites, scabies, pityriasis lichenoides, Langerhans cell histiocytosis, cutaneous B-cell lymphomas with immunoblastic or large-cell features, CD30+ large-cell transformation of mycosis fungoides (MR), LyP, and CD30+ cutaneous NK/T-cell lymphoma[13]. Exclusion of these diseases is essential before establishing a diagnosis of PC-ALCL. For example, CD30+ cells and CD4+ lymphocytes are traced in the infiltrates of LyP and PC-ALCL, whereas pityriasis lichenoides infiltrates demonstrate predominantly CD8+ lymphocytes[14].

The differential diagnosis between PC-ALCL and LyP might often be problematic. However, it is important, since patients with LyP have a 10–20% increased risk for developing lymphoid malignancy in comparison to PC-ALCL and because the therapeutic strategy among these conditions differs[9]. In general, individual lesions of LyP are usually <2 cm in diameter, whereas those of PC-ALCL exceed this dimension. While LyP and PC-ALCL of the skin appear as nodular lesions or tumors and have similar histopathological features, with large anaplastic CD30+ cells infiltrating the dermis, the distinction between the two is related to the depth of invasion of the infiltrate as well as to the clinical features and evolution of the disease in the patient. There are several markers used for the above-mentioned distinction, such as Bcl-2, fascin, CLA, and CD56. In LyP, Bcl-2 is absent, CD56 is often negative, CLA is highly expressed, and the percentage of the expression of fascin is 24%, while in PC-ALCL, Bcl-2 is positive in 30% of the cases, the positive expression of CD56 varies from 12 to 75%, CLA is variably expressed, and the percentage of the expression of fascin is 64%. Clonality, as demonstrated by T-cell receptor (TCR) gene rearrangement, is evident in all PC-ALCLs, but varies from 40 to 100% in LyP[5]. Furthermore, the lesions in LyP wax and wane, in contrast to those noted in PC-ALCL[6]. LyP is considered to be mainly an indolent disorder, with spontaneously healing skin papules and nodules occurring often over years; PC-ALCL may be indistinguishable from its more aggressive systemic counterpart, but it remains an indolent process with spontaneous regression in 25% of cases[14].

Finally, the distinction between PC-ALCL and MF is easy because of the presence of concurrent plaques, with the characteristic histology of MF in the latter case[3]. The conditions that must be considered in the differential diagnosis of PC-ALCL are highlighted in Table 1 and the comparison of clinicopathological characteristics of PC-ALCL, LyP, and systemic ALCL is shown in Table 2.

**TABLE 1**
**Conditions that Must be Considered in the Differential Diagnosis of PC-ALCL**

| |
|---|
| Systemic ALCL ALK+ |
| Systemic ALCL ALK- |
| Lymphomatoid papulosis |
| CD30+ large-cell transformation of mycosis fungoides |
| CD30+ cutaneous NK/T-cell lymphoma |
| Cutaneous B-cell lymphomas with immunoblastic or large-cell features |
| Langerhans cell histiocytosis |
| Pityriasis lichenoides |
| Arthropod bites |
| Scabies |

**TABLE 2**
**Comparison of Clinicopathological Characteristics of PC-ALCL, LyP, and Systemic ALCL**

| Criteria | PC-ALCL | LyP | Systemic ALCL ALK+ | Systemic ALCL ALK- |
|---|---|---|---|---|
| Clinical features | Older patients, median age 61 years | Median age 45 years | Children, young adults (median age third decade) | Adults (median age fifth decade) |
| | • Localized or generalized (multifocal) cutaneous manifestations<br>• Extracutaneous involvement in 5–10%<br>• Spontaneous resolution in 25% of patients | • Recurrent papulonecrotic eruption on extremities and trunk<br>• Hypo- or hyperpigmented scar<br>• Spontaneous regression in 4–6 weeks | • Lymph nodes and extranodal sites (skin, lung, liver, bone, bone marrow) | • Lymph nodes and extranodal sites (spleen, subcutaneous tissue, bone, bone marrow)<br>• Skin manifestations in 20% of patients |
| | 5-year survival 90% | Clinically benign, can progress to PC-ALCL, increased risk for MF and Hodgkin's lymphoma | 5-year OS by IPI: 4/5: 23% | 5-year OS by IPI: 4/5: 13% |
| Pathology | Cohesive sheets of CD30+ large cells with anaplastic, pleomorphic, or immunoblastic morphology | • Type A: large cells similar to Reed-Sternberg cells<br>• Type B: cerebriform cells that resemble MF<br>• Type C: large cells that resemble systemic ALCL | Cohesive sheets of CD30+ large cells (hallmark cells) | Cohesive sheets of CD30+ large cells (hallmark cells) |
| | • Variable expression of CLA, ALK expression rare, typically lack EMA<br>• Fascin+ (64%), Bcl-2+ (30%), CD56+ (12–75%)<br>• Few inflammatory cells; Reed-Sternberg-like cells | • CLA high, ALK absent, EMA absent<br>• Fascin+ (24%), Bcl-2 absent, CD56 often negative | • CLA low, ALK positive, EMA more often expressed (compared to systemic ALCL ALK-)<br>• Fascin+ (91%), Bcl-2+, CD56+ worse prognosis<br>• Broad morphologic spectrum; Reed-Sternberg cells absent | • CLA low, ALK negative, EMA less often expressed (compared to systemic ALCL ALK+)<br>• Fascin+ (91%), Bcl-2+, CD56+ worse prognosis<br>• Broad morphologic spectrum; Reed-Sternberg cells absent |

Abbreviations: ALCL: anaplastic large cell lymphoma; ALK: anaplastic lymphoma kinase; CLA: cutaneous lymphocyte antigen; EMA: epithelial membrane antigen; IPI: International Prognostic Index; MF: mycosis fungoides; OS: overall survival; PC-ALCL: primary cutaneous anaplastic large cell lymphoma. Combined data from Kadin[9] and Droc et al.[5].

## CASES

Several cases of PC-ALCL have been reported affecting, among other sites, the skin of eyelid and nose, mimicking keratoacanthomas[15], appearing after renal transplantation accompanied with neutrophil-rich, panniculitic lesions[16], or even arising from a burn scar, associated with vascular endothelial growth factor (VEGF)[17].

Another case has been reported where a patient presenting with an enlarging PC-ALCL mass on his left femoral region (8 × 10 cm) was treated by systemic chemotherapy, due to particular clinical course[18]. Finally, PC-ALCL with invasion of gastric mucosa 5 years after initial diagnosis and fatal leukemic progression has been described. This outcome challenges the initial diagnosis of PC-ALCL in the aforementioned case and raises the possibility of systemic ALCL (ALK-) involving the skin being the early disease[19].

Systemic ALCLs have been reported to infiltrate the eyelid[20], the myocardium[21], and the lung[22], or to present as cutaneous nodules in adolescents spreading to the central nervous system[21,22]. In the last three cases, the possibility of an initial PC-ALCL progressing to systemic ALCL with involvement of various organs is high.

## TREATMENT

Primary cutaneous lymphomas represent a broad spectrum of distinct entities with multiple clinical and pathologic presentations, prognoses, and treatment approaches. Given their rarity and heterogeneity, these entities represent diagnostic and therapeutic challenges, thus requiring a multidisciplinary approach and expertise to ensure appropriate diagnosis and management[14].

Patients with PC-ALCL have very good prognoses. The 5-year OS is >90%. As initial management, a follow-up for a period of 4–6 weeks is recommended, since 25% of PC-ALCL lesions will spontaneously regress. If the latter occurs, therapy is not indicated and observation is advised for possible disease recurrence. However, most patients have disease that does not regress spontaneously. In that case, the choice of therapy depends on two factors: (1) disease dissemination, and (2) the presence of localized or multifocal lesions. For a solitary lesion, local radiotherapy is the preferred treatment with a dose of 40 Gy. Electron-beam irradiation is preferred nowadays because of penetration only to the dermis. Thus, there are no systemic effects, even though side effects include local edema, atrophy of sweat glands, alopecia, and radiodermatitis. Surgical excision is an alternative approach for localized lesions and, associated with radiotherapy, leads to an excellent prognosis. Importantly, surgical excision specimens may contain margins contaminated by the disease[9].

Single-agent therapy using low-dose methotrexate is effective for patients with multifocal disease or for those who do not respond well to surgical and radiation treatment. The dose of 15–20 mg weekly is followed by response in approximately 87% of the patients. The duration of treatment with methotrexate is unknown for patients with multifocal PC-ALCL. For patients who respond initially to methotrexate, a conservation period of 1–4 weeks is recommended, provided there is no liver involvement. Care should be taken to avoid long-term treatment with methotrexate to avoid complications like hepatic fibrosis. Furthermore, methotrexate is not recommended for women of childbearing age or for those with blood dyscrasias[9,23,24].

Another therapeutic approach for patients whose disease is refractory on methotrexate is oral etoposide, which has been shown to be safe and effective[25]. Interferon-γ is also another possibility for treating PC-ALCL. Alternative therapies, which can be administered only in selected cases, include among others, the purine nucleoside analog pentostatin, retinoids, oral bexarotene, interferon-α, interleukin-12, and imiquimod[23,26]. Moreover, targeting of radiation, drugs, and protein toxins to cancers selectively with monoclonal antibodies (MAbs) has been a topic of considerable interest and an area of continued development[27]. In this regard, the monoclonal antibody against CD30 is a promising approach for the treatment of PC-ALCL[28].

Patients presenting with multifocal skin lesions and/or regional lymph nodes have prognoses similar to those of patients with skin lesions only. Thus, regional lymph node involvement does not represent a poor prognostic factor. Nevertheless, patients with enlarged distant lymph nodes, disseminated skin disease, extracutaneous involvement, and systemic organ participation have a worse prognosis and should be treated with multiagent chemotherapy. Many clinicians, however, are unaware of the spectrum of CD30+ PCLPDs and patients have been misdiagnosed or treated with unnecessarily aggressive regimens. Moreover, it is important to distinguish the benign from the potentially malignant or progressive entities[29].

In contrast, patients with disseminated skin disease seem to be at greater risk of developing extracutaneous involvement and should be considered for multiagent chemotherapy, similar to that used in the treatment of systemic ALCL, such as the cyclophosphamide, doxorubicin, vincristine, and prednisone (CHOP) regimen. However, the number of cycles has not been specified[23,25]. Moreover, it is difficult to administer doxorubicin to the elderly because of its cardiotoxicity. Therefore, the use of the regimen VNCOP-B, which includes etoposide, mitoxantrone, cyclophosphamide, vincristine, prednisone, and bleomycin, is useful since it is characterized by low incidence of cardiotoxicity and mucosal symptoms[30]. Finally, high-dose chemotherapy followed by stem-cell rescue may be indicated in affected high-risk patients[9].

Treatment of choice in patients with PC-ALCL should rely on the extent of skin and/or extracutaneous disease. Aggressive, multiagent chemotherapy is rarely indicated. Moreover, chemotherapy given to patients with PC-ALCL is usually not curative and early relapse is common, so it is important to make the diagnosis of skin vs. systemic lymphoma. Appropriate staging for all newly diagnosed patients with CD30+ PCLPDs includes imaging studies of lymph nodes and visceral sites to exclude systemic involvement. The role of bone marrow biopsy is less well defined. Bone marrow is rarely involved in patients with PCLPDs and may be deferred except in patients who demonstrate evidence of extracutaneous involvement[14]. It is essential to monitor patients with an initial PC-ALCL diagnosis for potential dissemination or extracutaneous spread of the disease, which includes lymph node or systemic organ involvement. Furthermore, such patients might face a relapse in their disease or even develop other malignancies, such as MF, Hodgkin, or non-Hodgkin lymphoma[9]. In conclusion, a multidisciplinary approach with dermatology, radiation oncology, and medical oncology is essential and should be preferred.
